# Low-normal *FMR1* CGG repeat length: phenotypic associations

**DOI:** 10.3389/fgene.2014.00309

**Published:** 2014-09-09

**Authors:** Marsha R. Mailick, Jinkuk Hong, Paul Rathouz, Mei W. Baker, Jan S. Greenberg, Leann Smith, Matthew Maenner

**Affiliations:** ^1^Waisman Center, University of Wisconsin–MadisonMadison, WI, USA; ^2^Department of Biostatistics and Medical Informatics, University of Wisconsin–MadisonMadison, WI, USA; ^3^Wisconsin State Laboratory of HygieneMadison, WI, USA; ^4^School of Social Work, University of Wisconsin–MadisonMadison, WI, USA

**Keywords:** *FMR1* CGG expansions, fragile X syndrome, genotype–phenotype correlations

## Abstract

This population-based study investigates genotype–phenotype correlations of “low- normal” CGG repeats in the fragile X mental retardation 1 (*FMR1*) gene. *FMR1* plays an important role in brain development and function, and encodes FMRP (fragile X mental retardation protein), an RNA-binding protein that regulates protein synthesis impacting activity-dependent synaptic development and plasticity. Most past research has focused on CGG premutation expansions (41–200 CGG repeats) and on fragile X syndrome (200+ CGG repeats), with considerably less attention on the other end of the spectrum of CGG repeats. Using existing data, older adults with 23 or fewer CGG repeats (2 SDs below the mean) were compared with age-peers who have normal numbers of CGGs (24–40) with respect to cognition, mental health, cancer, and having children with disabilities. Men (*n* = 341 with an allele in the low-normal range) and women (*n* = 46 with two low-normal alleles) had significantly more difficulty with their memory and ability to solve day to day problems. Women with both *FMR1* alleles in the low-normal category had significantly elevated odds of feeling that they need to drink more to get the same effect as in the past. These women also had two and one-half times the odds of having had breast cancer and four times the odds of uterine cancer. Men and women with low-normal CGGs had higher odds of having a child with a disability, either a developmental disability or a mental health condition. These findings are in line with the hypothesis that there is a need for tight neuronal homeostatic control mechanisms for optimal cognitive and behavioral functioning, and more generally that low numbers as well as high numbers of CGG repeats may be problematic for health.

## INTRODUCTION

The fragile X mental retardation 1 (*FMR1*) gene plays an important role in brain development and function (Brown, 2002). This gene encodes FMRP (fragile X mental retardation protein), an RNA-binding protein that regulates protein synthesis impacting activity-dependent synaptic development and plasticity (Bassell and Warren, 2008).

The full mutation of the gene, a trinucleotide (CGG) repeat expansion, results in fragile X syndrome, which occurs when there are more than 200 CGG repeats in the 5^′^ untranslated region of the *FMR1* gene, resulting in the gene becoming fully methylated and thus silenced. The premutation and gray zone are defined, respectively, as 55–200 CGG repeats, and 45–54 CGG repeats, according to the American College of Medical Genetics (Maddalena et al., 2001). Some recent studies have used a lower boundary to define the beginning of the gray zone (e.g., 41 CGG repeats in Hall et al., 2011). There has been intense interest in *FMR1* CGG expansions from the perspectives of basic science, genotype–phenotype correlations, epidemiology, and public policy. However, very little attention has been focused on the other end of the spectrum, namely smaller than normal numbers of CGG repeats. The purpose of the present paper is to present descriptive data on genotype–phenotype correlations on what has been termed “low-normal” numbers of CGG repeats (Chen et al., 2003).

Studies of *FMR1* CGG repeats have reported a wide normal range, with the modal number of repeats being 30 (Fu et al., 1991; Chen et al., 2003). While epidemiological studies have estimated the prevalence of CGG expansions (Seltzer et al., 2012; Tassone et al., 2012; Maenner et al., 2013), low numbers of CGG repeats have been reported in only several studies and no epidemiological studies have yet been conducted. For example, Fu et al. (1991) reported that six CGG repeats was the lowest in the collection of samples they analyzed. Kremer et al. (1991) concluded that the normal gene has 40 ± 25 CGG repeats, and by this standard as few as 15 CGG repeats would be considered to be in the normal range. Snow et al. (1993) characterized the CGG repeat at the *FMR1* locus in more than 700 individuals, with the lowest repeat length reported to be 13 CGGs. Wang et al. (2013) studied healthy adult males, with the lowest number of CGG repeats found to be 19.

Chen et al. (2003) reported that the efficiency of translation was a function of the number of CGG repeats, with the modal number of 30 repeats conferring the greatest efficiency of translation. Their data showed that having *fewer* or *greater* numbers of CGG repeats reduced the efficiency of the translation. With respect to the low end of the distribution, they observed that the efficiency of translation increased by nearly twofold as the numbers of CGG repeats increased from 0 to 30. This observation suggests a possible clinical phenotype associated with low numbers of CGG repeats, and also that there may be some similarities between the clinical manifestations of both low numbers of CGG repeats and expansions, because both are associated with inefficient translation.

A clinical report of two cases with duplications of the *FMR1* gene and two cases with deletion of *FMR1* showed that “both loss and gain of *FMR1* copy number can lead to overlapping neurodevelopmental phenotypes” (Nagamani et al., 2012, p. 333). Ramocki and Zoghbi (2008) articulated the necessity of tight neuronal homeostatic control mechanisms for normal cognition and behavior, and suggested that neurodevelopmental and neuropsychiatric disorders may in part be the result of imbalances in homeostatic controls in multiple genes, including *FMR1*.

Two other studies offer clues about possible effects of low-normal numbers of CGG repeats in the *FMR1* gene. Wang et al. (2013) reported the influence of *FMR1* on working memory and brain structure in normal males. Although CGG repeat length was not directly associated with working memory or brain structure in this sample, *FMR1* mRNA and FMRP were significant correlates. The study findings suggest that lower levels of gene expression, even within the normal CGG repeat range, have negative effects on cognition.

Low CGG repeats in the *FMR1* gene have also been implicated in research on reproductive biology. Weghofer et al. (2012) cross-tabulated the co-occurrence of *BRCA1/2* mutations and *FMR1* repeat length distribution, and observed that *BRCA1/2* carriers almost invariably had low numbers of CGG repeats in their *FMR1* gene (<26 CGGs), compared to controls. They inferred that *BRCA1/2* mutations are embryo-lethal unless rescued by low CGG repeats in the *FMR1* gene, and predicted that women with low *FMR1* CGG repeats should have an increased risk of *BRCA1/2*-associated cancers.

The present study is a secondary analysis of an existing population-based, non-clinical sample that integrates phenotypic information with genetic data including *FMR1* CGG repeat length. We identified 341 men who fell into the low-normal category of CGG repeats (see below for the definition of low-normal). We also identified 46 women for whom *both* alleles were in the low-normal category. We examined specific characteristics of these individuals with low numbers of CGG repeats in the *FMR1* gene and whether they differed from those who have normal numbers of CGG repeats. We carefully selected the study variables to reflect domains implicated in past research to be correlated with low numbers of CGG repeats, namely cognition (Ramocki and Zoghbi, 2008; Wang et al., 2013), mental health (Ramocki and Zoghbi, 2008), and cancers of the breast and uterus (Weghofer et al., 2012). Since the numbers of CGG repeats in the *FMR1* gene are passed from parent to child, we also examined whether low numbers of CGG repeats would be associated with elevated odds of having children with neurocognitive or neuropsychiatric disabilities, as these data were also available.

## MATERIALS AND METHODS

### WISCONSIN LONGITUDINAL STUDY

Data were obtained from the Wisconsin Longitudinal Study (WLS), a random sample of 10,317 women and men who graduated from Wisconsin high schools in 1957, representing one-third of that age cohort (Hauser et al., 1998). In 1957, 75% of Wisconsin 18 year olds were high school graduates. Follow-up studies were conducted in 1975 with 9,138 (90.1%) surviving members of the original sample when they were, on average, 36 years old; in 1992 with 8,493 (87.2%) of the surviving respondents when they were in their early 50 s; and in 2004 with 7,265 (80.0%) of the surviving respondents when they were in their mid-60 s; and in 2011 with 5,969 (68.4%) of the surviving respondents when they were 71 years of age. In addition, parallel data collection procedures were conducted with one randomly selected sibling of a subset of the respondents in 1977, 1994, 2005, and 2011, with 5,823 siblings participating in one or more of these data collection points. The original respondents were members of a single cohort (high school graduates in the year 1957), but their siblings ranged from age 46 to 92 when the data reported here were collected (62.4% of the entire sample was age 71 or 72 at that time).

Although all of the original WLS participants were high school graduates, as were 93% of their siblings, WLS participants ranged in IQ score (as measured during high school) from a low of 61 (the floor of the test, described below) to a high of 145. Fifteen percent had IQ scores of 85 (1 SD below the mean) or below. This percentage is approximately the expected proportion of the population on the low end of the IQ distribution (16% of the population is expected to be 1 SD below the mean or lower). Reflecting Wisconsin’s population at the mid-20th century, the WLS sample is racially and ethnically homogeneous; 99.2% are White (84.2% of northern or central European heritage).

In 2006 and 2007, WLS collected saliva samples from participants using Oragene kits (DNA Genotek, Inc.) and a mailback protocol patterned closely on a previous Swedish study (see Rylander-Rudqvist et al., 2006). Oragene kits were selected because of their ability to be used in a mailback protocol (e.g., no need for immediate freezing) and their high average DNA yield (in our sample, median = 319 μg/mL, mean = 400 μg/mL, SD = 284 μg/mL). All participants provided informed consent under a protocol approved by the Institutional Review Board of the University of Wisconsin-Madison. 56% of WLS participants alive in 2006 provided saliva samples (*n* = 7044). Those who sent saliva had one-half year more schooling (13.9 years vs. 13.4 years, *p* < 0.001), three points higher IQ scores (103.2 vs. 99.5, *p* < 0.001), and higher high school rank (54.6 vs. 46.7, *p* < 0.001) than those who did not return saliva samples. Otherwise, they were representative of the WLS sample as a whole.

Of the 7044 saliva samples, 15 were not used for the present analysis because of ambiguous sex determination. Another 297 were not used because there was insufficient DNA for the CGG repeat assay. Therefore, the present study is based on 6732 cases whose saliva sample yielded sufficient DNA for the CGG repeat assay, of whom 3263 (48.5%) were males and 3469 (51.5%) were females. These cases included 1311 sibling pairs.

### DETERMINATION OF THE *FMR1* CGG TRIPLET REPEAT NUMBER

The number of *FMR1* CGG repeats was determined for all biological samples using a PCR-based protocol that incorporated reagents developed and manufactured by Celera Corporation. The specific procedures we used were described previously (Seltzer et al., 2012). The protocol combined gene-specific primers that flank the CGG repeat region of the *FMR1* gene with gender-specific primers, a polymerase mixture, and a reaction buffer that is optimized for amplification of GC-rich DNA. Besides CGG repeat data, this assay also detects the presence of X and Y chromosomes within a sample, enabling sex confirmation and identifying female samples with a single detectable CGG repeat (apparent homozygosity). Data were analyzed using GeneMapper^®^; v. 4.0 (Applied Biosystems). CGG triplet repeats were calculated using the following formula: number of CGG repeats = (peak size–193)/3.

### CLASSIFICATION OF LOW-NORMAL CGG REPEATS

We defined the upper boundary of low-normal CGG repeats based on the distribution of CGG repeats in the WLS population. The mean was 30.6 CGGs, with a standard deviation of 3.8. Two standard deviations below the mean was 23 CGG repeats or fewer. For males, their *FMR1* gene on the X chromosome had to have 23 or fewer CGG repeats to be classified into the low-normal group. Otherwise, they were classified as having normal CGG repeats (i.e., 24–40 CGG repeats). Males with 41 or more CGG repeats were omitted from the present analysis (*n* = 164).

For females, both of their *FMR1* alleles had to have 23 or fewer CGG repeats to be included in the low-normal group. Females with 24–40 CGG repeats on both alleles were classified as having normal-length CGG repeats (*n* = 2452). Omitted from the present analysis were females with any gray zone or premutation-length CGG repeats (*n* = 295), as well as those who had one low-normal and one normal CGG repeats (*n* = 676). For females, we dropped those with one normal and one low-normal allele because we did not have access to X-inactivation data, and thus it was not possible to determine the proportion of normal alleles vs. the proportion of low-normal alleles that were active for any given sample member.

Based on these definitions, 341 males (11%) and 46 females (1.8%) were classified as having low-normal CGG repeats and 2758 males and 2452 females were classified as having normal-length CGG repeats. For males, the lowest number of CGG repeats in this sample was 9, whereas for females, the lowest was 11 CGG repeats.

### MEASUREMENT OF DEMOGRAPHIC AND PHENOTYPIC CHARACTERISTICS

Three rounds of data from the WLS were used to measure the variables for the present study: demographic data collected in 1957 and 2011, identification of number of children and children with disabilities in the 2004/06 and 2011 rounds of data collection, and outcome data collected in 2011, when respondents averaged 71 years of age.

For the present study we included three variables from the 1957 data: the years of education completed by respondent’s father and mother, and the respondent’s IQ score as measured in high school. Scores on the Henmon-Nelson Test of Mental Ability (Henmon and Nelson, 1937), administered at age 17, were available for all WLS respondents. As noted, IQ scores ranged from 61 to 145 (mean = 103.3, SD = 15.0). The test measures a wide variety of mental abilities, including verbal, spatial, and numerical knowledge and reasoning, the composite of which reflects generalized intellectual functioning. Reliability coefficients for the Henmon-Nelson are consistently around 0.90 (Buros, 1940). The scores on the original version of the test show good predictive validity in forecasting academic success in college (Drake and Henmon, 1937), and scores on a later revision were correlated 0.83 with IQs from an individually administered test of intelligence (Wagner, 1981). Within the WLS sample, Henmon-Nelson IQs were correlated 0.60 with high school grade rank, which is consistent with the usual associations found between IQ and school achievement (Gregory, 2004).

Other demographic characteristics used to describe the sample for the current study were years of education completed by the respondent, household income of the respondent, and marital status (1 = currently married, 0 = not currently married). These demographic characteristics reflect status as of the 2011 round of data collection.

We compared those with normal and low-normal CGG repeats with respect to outcome variables in the following domains: cognition, mental health, cancer, and having children with disabilities. *Cognition* was measured in 2011 by the HUI Mark 3 cognition score (Feeny et al., 1996), which has six levels of cognitive functioning based on respondents’ answers to two questions: “how would you describe your ability to remember things?” and “ how would you describe your ability to think and solve day to day problems?” The six levels of cognitive score are: 1 = able to remember most things, think clearly and solve day to day problems; 2 = able to remember most things, but have a little difficulty when trying to think and solve day to day problems; 3 = somewhat forgetful, but able to think clearly and solve day to day problems; 4 = somewhat forgetful, and have a little difficulty when trying to think or solve day to day problems; 5 = very forgetful, and have great difficulty when trying to think or solve day to day problems; and 6 = unable to remember anything at all, and unable to think or solve day to day problems. The majority (60.8%) were in the least impaired category of this measure, and less than 1% classified themselves in the most impaired category.

*Mental health* was measured by anxiety, depression, and alcohol symptoms. Anxiety was measured by the Spielberger Anxiety Index (Spielberger et al., 1970), which is a summary score of seven items asking the number of days during the past week respondents felt each emotional state: calm, tense, being at ease, worrying over possible misfortune, nervous, jittery, and relaxed. For depression, respondents reported whether since the previous WLS interview (an average of 6 years previously), they experienced a period of 2 weeks or more when nearly every day they felt sad, blue, depressed, or when they lost interest in most activities, such as work, hobbies, or things they usually liked to do for fun (1 = yes, 0 = no). Episodes of depression associated with physical illness, medications, and alcohol or drug use were not included in this measure. For alcohol symptoms, respondents reported whether they had to drink more to get the same effect as when they first started drinking (1 = yes, 0 = no). This is a standard item from the DSM used to determine whether an individual has developed a tolerance for alcohol and the effect refers to the feeling the individual is trying to achieve by drinking (typically a feeling of intoxication).

*Cancer* (for women) included lifetime history of breast cancer (coded 1 if yes and 0 if no) and uterine cancer (similarly coded).

For women, having *children with disabilities* was coded 1 if the respondent had a biological child with a developmental disability or mental health condition, and 0 if not. For men, the analysis of this variable was restricted to fathers of daughters, as it is only daughters who inherit a father’s X chromosome. Respondents who had children with disabilities were identified through a series of screener questions asked during the 2004/06 and 2011 rounds of data collection. The screener consisted of a maximum of 31 questions that began by asking parents if any of their children (living or deceased) had an intellectual or developmental disability and the specific diagnosis. If the parent indicated a specific condition (e.g., Down syndrome, fragile X syndrome, autism), or used terms such as developmental disability, mental retardation, or cognitive disability, he or she was coded as having a child with a disability. In a small number of cases (5.4%), the parent did not know the specific diagnosis given to his or her child, but indicated that the child had difficulties in school. In such cases, branching follow-up questions asked if the child was below-average in intelligence, attended special education classes, and/or had difficulty performing activities of daily living. If so, he or she was classified as having a disability. Other childhood disabilities mentioned by the respondents were noted, such as attention deficit hyperactivity disorder, seizure disorder, etc., and included in this measure of having a child with a disability. Parents also reported whether a child had been diagnosed by a health professional with schizophrenia, bipolar disorder, or a major clinical depression (that required hospitalization or limited the ability of the person to carry on activities of daily living). Suicide in a child was based on parental report of cause of death. Other mental health diagnoses mentioned by parents, such as anxiety disorders, were noted and included in this measure of having a child with a disability.

### STATISTICAL ANALYSIS

For each outcome variable, we estimated a regression model, including controls for age and sex, and the category of CGG repeat (normal or low-normal). We controlled for age and sex because of known associations between these variables and several of the outcome variables (e.g., cognition, health) and because of the wide age range of the sample (46 to 92 in 2011). We used the generalized estimating equation (GEE; Diggle et al., 2013) approach to regression modeling, and report regression coefficients (for continuous outcome variables) or odds ratios (for binary outcome variables) with robust standard errors and 95% confidence intervals based on clustering at the level of sibling pair. We also tested the Sex X CGG repeat category interaction effect. We report coefficients or odds ratios for age and sex from the models without the interaction. If the interaction was not significant, we also report the CGG repeat effect from this same model. If the interaction was significant, it is reported, and separate CGG effects are reported for males and females. Use of the GEE modeling approach was motivated by the study design, which as noted earlier, included 1311 sibling pairs whose endpoints on the outcome variables might be correlated.

Due to variable amounts of missing data across outcome variables, sample sizes for males in the low-normal CGG repeat category ranged from 304 to 341, and in the normal CGG repeat category ranged from 2418 to 2690. For females, sample sizes in the low-normal CGG repeat category ranged from 42 to 46, and in the normal CGG repeat category from 2213 to 2404.

## RESULTS

### DEMOGRAPHIC CHARACTERISTICS

In the aggregate, participants in the present study with low-normal CGG repeat lengths did not differ significantly from those who had normal length CGG repeats with respect to any of the background demographic characteristics. As shown in **Table 1**, their fathers and mothers had similar levels of education (between 9 and 11 years). When in high school, the two groups of participants had equivalent standardized IQ score measurements (between 103 and 106). The two groups had almost identical years of education (13–14 years). They did not differ in their number of biological children (ranging from 2.5 to 2.8).

**Table 1:** Descriptive statistics by CGG repeat category and sex.

	Males		Females	
	Low-Normal^1^	Normal^2^	Low-Normal^3^	Normal^4^
	Mean (s.d.)	Mean (s.d.)	Mean (s.d.)	Mean (s.d.)
**Demographic variables**
Age in 2011 (years)	71.2 (4.0)	71.2 (4.1)	70.4 (4.2)	71.0 (4.1)
Years of education – Father	10.1 (3.4)	10.0 (3.5)	9.1 (3.5)	9.8 (3.5)
Years of education – Mother	10.8 (3.1)	10.7 (2.8)	10.9 (3.1)	10.6 (2.8)
Years of education – Respondent	14.4 (2.6)	14.3 (2.6)	13.6 (2.2)	13.6 (2.2)
IQ score	103.5 (16.1)	103.0 (15.4)	105.8 (14.6)	103.5 (14.7)
Number of biological children	2.53 (1.57)	2.57 (1.53)	2.67 (1.70)	2.76 (1.63)
Marital status (2011; 1 = married, 0 = other)	0.832 (0.374)	0.842 (0.364)	0.643 (0.484)	0.660 (0.473)
Household income in dollars (2011)^5^	43030 (46550)	40800 (44948)	29304 (34612)	28874 (28754)
**Outcome variables**
Cognition (1 = least problems to 6 = most problems)	2.03 (1.26)	1.86 (1.18)	1.83 (1.27)	1.80 (1.13)
Anxiety	6.24 (6.95)	6.22 (7.19)	9.93 (9.95)	7.54 (7.54)
Depressive episode in last 6 years (1 = yes, 0 = no)	0.032	0.049	0.192	0.102
Need to drink more for the same effect (1 = yes, 0 = no)	0.133	0.123	0.192	0.039
Breast cancer (1 = yes, 0 = no)	n/a	n/a	0.195	0.091
Uterine cancer (1 = yes, 0 = no)	n/a	n/a	0.049	0.013
Has biological child with disabilities (1 = yes, 0 = no)^6^	0.068	0.044	0.219	0.115

*^1^Sample size ranges from 304 to 341; ^2^Sample size ranges from 2418 to 2690; ^3^Sample size ranges from 42 to 46; ^4^Sample size ranges from 2213 to 2404; ^5^Median income is reported with interquartile range in parenthesis; ^6^Based on females who had at least one biological child and males who had at least one biological daughter.*

At an average age of 71, those who had low-normal numbers of CGG repeats did not differ from those with normal numbers of CGG repeats in current marital status (with 83 to 84% of males and 64 to 66% of females currently married) or household income.

### DIFFERENCES IN OUTCOME VARIABLES BETWEEN THOSE WITH LOW-NORMAL CGGS vs. NORMAL CGG REPEATS

**Table 1** also shows the means and standard deviations for the outcome variables, broken down by low-normal and normal CGG categories. **Table 2** presents the results of the regression models that tested whether and how those in the low-normal CGG repeat category differed from those in the normal category with respect to the outcome variables. All models adjusted for age. In models in which the Sex X CGG repeat category interaction term was non-significant, the regression models also adjusted for sex. In models with a significant interaction term, differences in the outcome variables were reported separately by sex.

**Table 2:** Generalized estimating equation analysis of CGG repeat category and outcome variables^**1**^.

	Cognition	Anxiety	Recent depressive episode (1 = yes, 0 = no)	Need to drink more for the same effect (1 = yes, 0 = no)	Breast cancer (1 = yes, 0 = no)	Uterine cancer (1 = yes, 0 = no)	Child with disabilities^2^ (1 = yes, 0 = no)
	b^3^ (s.e.)^4^ [95% CI]	b (s.e.) [95% CI]	Odds ratio (s.e.) [95% CI]	Odds ratio (s.e.) [95% CI]	Odds ratio (s.e.) [95% CI]	Odds ratio (s.e.) [95% CI]	Odds ratio (s.e.) [95% CI]

Age (years)	0.02 (0.00)*** [0.01, 0.03]	-0.07 (0.03)* [-0.13, -0.01]	0.98 (0.01) [0.96, 1.01]	0.95 (0.01)*** [0.92, 0.97]	1.06 (0.02)** [1.02, 1.09]	0.97 (0.04) [0.91, 1.05]	0.98 (0.01) [0.95, 1.00]
Sex (female = 1)	-0.07 (0.03)* [-0.14, -0.01]	1.39 (0.23)*** [0.93, 1.83]	1.71 (0.18)*** [1.39, 2.09]	0.31 (0.04)*** [0.23, 0.41]	–	–	1.42 (0.17)** [0.02, 0.76]
CGG repeat (low repeat = 1)	0.15 (0.07)* [0.01, 0.29]	0.35 (0.46) [-0.55, 1.25]	0.91 (0.28) [0.49, 1.67]	(For males) 1.08 (0.23) [0.71, 1.64] (For females) 5.99 (3.13)*** [2.14, 16.7]	2.53 (1.02)* [1.15, 5.61]	3.73 (2.75)+ [0.88, 15.82]	1.68 (0.34)* [1.12, 2.50]
CGG repeat x sex	–	-	-	5.53 (3.12)** [1.82, 16.7]	-	-	-

p ≤ 0.10, *p ≤ 0.05, **p ≤ 0.01, ***p ≤ 0.001.

*^1^Generalized estimating equation coefficients (or odds ratio for binary dependent variables) with robust standard errors and 95% confidence intervals are reported.*

*^2^The sample for the analysis was restricted to females who had at least one biological child or males who had at least one biological daughter (i.e., the outcome is a probability of having a biological child with a non-normative condition, given that a respondent had at least one biological child, or one biological daughter in the case of males).*

*^3^“b” refers to the regression coefficient.*

*^4^“s.e.” refers to standard error.*

#### Cognition

Sample members with low-normal numbers of CGGs had more compromised cognitive functioning than those with normal numbers of CGG repeats, reflecting that those with low-normal numbers of CGG repeats reported more difficulty “remembering things and thinking and solving day to day problems” during the past 4 weeks than those with normal numbers of CGGs (*p* < 0.05). Note that although those with low numbers of CGGs reported greater cognitive difficulties in later life, they did not differ in IQ score as measured when they were in high school (see **Table 1**).

#### Mental health

**FIGURE 1:**
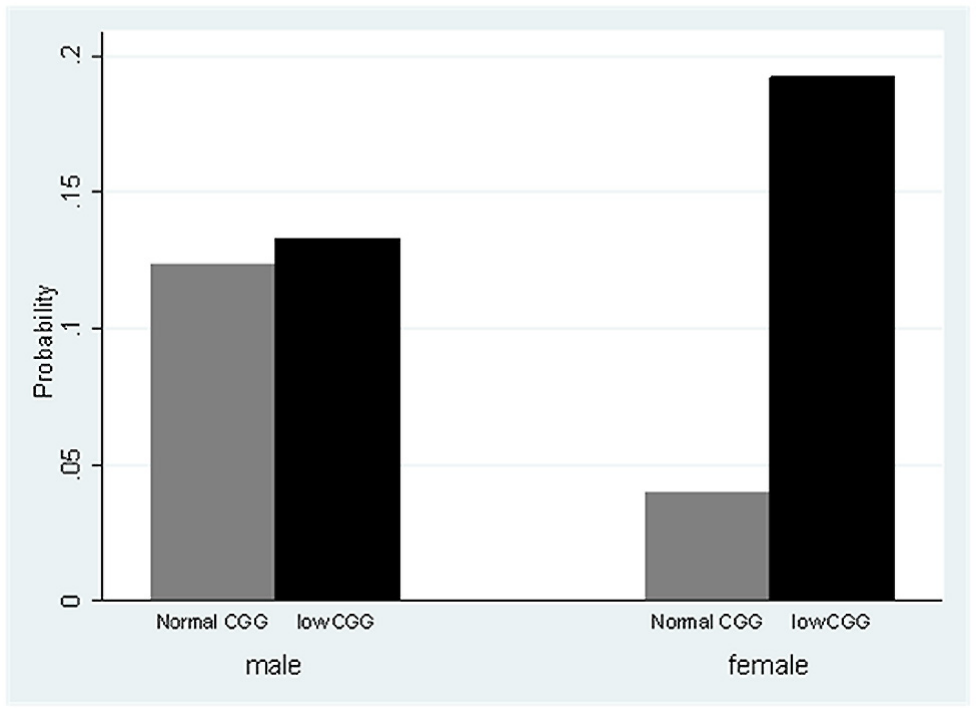
**Estimated probability of needing to drink more for the same effect, by CGG group and sex**.

There was no detected effect of CGG repeat category on anxiety or depression. There was a significant Sex X CGG repeat category interaction (*p* < 0.01) with respect to the self-reported perception of needing to drink more to have the same effect (see **Figure 1**). Females in the low-normal CGG repeat category had nearly six times the odds of needing to drink more to have the same effect than females in the normal CGG repeat category (odds ratio = 5.99, *p* < 0.001), whereas for males, there was no difference between those in the low-normal vs. normal CGG repeat category.

#### Cancer

Women in the low-normal CGG repeat category had two and a half times the odds of having had breast cancer than those in the normal repeat category (odds ratio = 2.53, *p* < 0.05), and at a trend level had almost four times the odds of having had uterine cancer (odds ratio = 3.73, *p* = 0.074).

#### Having a child with disabilities

Respondents in the low-normal CGG repeat category had significantly greater odds of having had a child with a disability than those in the normal CGG repeat category (odds ratio = 1.68, *p* < 0.05). **Table 3** lists the types of disabilities of the children whose parents had low-normal CGG repeats vs. those whose parents had normal numbers of CGG repeats. Due to the small numbers of each type of disability, we did not carry out statistical tests of differences in prevalence of specific categories between the low-normal and normal CGG repeat group, but inspection of the data suggests that the apparent excess in the low-normal CGG category reflected higher numbers of children with ADHD/LD/seizures, intellectual disability, bipolar disorder, and suicide.

**Table 3:** Conditions of children with disabilities.

Disability condition	Normal CGGs^1^	Low-normal CGGs^2^
No disability	3884 (91.93%)	280 (91.20%)
ADHD, LD, seizures	68 (1.61%)	8 (2.61%)
Intellectual disability	22 (0.52%)	4 (1.30%)
Autism	7 (0.17%)	0
Genetic DD syndromes	3 (0.07%)	0
Cerebral palsy	12 (0.28%)	0
Down syndrome	7 (0.17%)	0
Brain injury	3 (0.07%)	0
Other DD	8 (0.19%)	0
Bipolar disorder	109 (2.58%)	10 (3.26%)
Depression	49 (1.16%)	2 (0.65%)
Schizophrenia	20 (0.47%)	0
Suicide	7 (0.17%)	2 (0.65%)
Other MI	26 (0.62%)	1 (0.33%)

*^1^341 sample members [of 4225, or 8.1% of sample members with at least one biological child (for females) or at least one biological daughter (for males)] with normal numbers of CGG repeats had a child with a disability.*

*^2^27 sample members [of 307, or 8.8% of sample members with at least one biological child (for females) or at least one biological daughter (males)] with low-normal numbers of CGG repeats had a child with a disability.*

## DISCUSSION

In line with the hypothesis that there is a need for tight neuronal homeostatic control mechanisms for optimal cognitive and behavioral functioning (Ramocki and Zoghbi, 2008), we report descriptive data to suggest that having numbers of CGG repeats on the low end of the normal distribution may be associated with poorer outcomes. Older men and women with CGG repeats at least two standard deviations below the mean had significantly more difficulty with their memory and ability to solve day to day problems. Women with both *FMR1* alleles in the low-normal category had significantly elevated odds of feeling that they need to drink more to get the same effect as in the past. These women also had elevated odds of breast and uterine cancer. Men and women with low-normal CGGs had higher odds of having a child with a disability, either a developmental disability or a mental health condition. However, due to the small sample size in the present study of individuals with low-normal CGGs (particularly women), these findings need to be interpreted with caution.

The differences between groups that were found across domains may be interrelated and thus may not be independent effects. For example, a diagnosis of and treatment for breast or uterine cancer has been associated with cognitive difficulties (Burgess et al., 2005). Having a child with a disability has been shown to be associated with elevated stress (Seltzer et al., 2011; Bailey et al., 2012), which might lead to excess alcohol consumption. It is also possible that excess alcohol consumption may increase the risk of cognitive difficulties. However, it also possible that some or all of the outcomes were the direct effects of low numbers of CGGs in the *FMR1* gene, with downstream effects on cognition and behavior. In the case of increased odds of cancer, past research suggested that having low numbers of CGGs rescued embryos carrying the *BRCA1/2* mutation, an example of a direct biological mechanism. The interrelatedness of the outcomes associated with low-normal CGGs, as well as the mechanism by which these outcomes emanate from low numbers of CGGs warrants investigation in future research.

Certain characteristics of the WLS make it a particularly useful resource for exploring the effect of low-normal CGG repeats in the *FMR1* gene. It is a non-clinical study, and the sample members were randomly selected from the population. Therefore, the results were not based on clinic participants or study volunteers, nor were they from members of families known to have abnormalities in the *FMR1* gene. Thus, the biases that may be introduced by these factors were avoided in the present sample. However, because DNA was not obtained until 2006, attrition due to early death or dropping out of the study limited the size of the sample for the present analysis.

Another characteristic of the WLS was that nearly all study participants were White, reflecting Wisconsin’s population in the mid-20th century. Previous research (Gleicher et al., 2010) reported that White females had higher rates of abnormal CGG repeats (either higher or lower than the normal range) than females of African or Asian descent, and in the Gleicher study, it was only in White females where both alleles were abnormal.

Additionally, WLS collected the data for the outcome variables that we examined in this study when participants were older adults (mean age = 71). It is known that *FMR1* CGG *expansions* result in an increasing phenotype as adults age. For example, symptoms of fragile X-associated tremor/ataxia syndrome (FXTAS) become evident after the age of 50 in some men and fewer women with premutation expansions (Hagerman and Hagerman, 2004), and an age-associated pattern of worsening linguistic dysfluency is also evident in women with premutation expansions (Sterling et al., 2013). It is similarly possible that the effects of *low numbers* of CGG repeats may not manifest until older age. The demographic data we reported on the WLS participants, collected earlier in their lifespan, suggest a similar age-associated pattern of low-CGG effects, as study participants with low-normal CGGs did not have a lower IQ score in high school or fewer years of education than those with normal numbers of CGG repeats, but in older age they did have more difficulties with cognition. The lifetime risk of cancer is best measured later in life, again revealing why the WLS is a particularly unique and valuable resource for this preliminary investigation.

However, the study has several limitations. In addition to the small sample size, the present findings should not be generalized to non-white racial and ethnic groups, given the known reduction in prevalence of both expansions and low numbers of CGG repeats in the *FMR1* gene. Furthermore, direct clinical phenotyping of individuals with low numbers of CGG repeats would be needed to confirm the patterns in the secondary analysis of data reported here, as the data were collected for other purposes. A related limitation is that the phenotypic data were based on self-reports. Mechanistic investigations are needed to identify direct effects of low CGGs and to differentiate them from secondary effects. In addition, future research that tracks intergenerational transmission of low numbers of CGG repeats would be extremely valuable. It is unfortunate that the WLS did not collect DNA from the children of participants. Such data could have provided a deeper understanding of the elevated risk of child disability in the next generation of study participants with low numbers of CGG repeats, as well as the stability of transmission of low-normal CGG repeats.

In conclusion, this preliminary investigation suggests that low-normal numbers of CGG repeats may have substantial implications for cognitive functioning, cancer, and the odds of having children with neurodevelopmental or neuropsychiatric conditions. Larger epidemiological studies as well as biologically based mechanistic investigations are necessary next steps.

## Conflict of Interest Statement

The authors declare that the research was conducted in the absence of any commercial or financial relationships that could be construed as a potential conflict of interest.
